# Trans-Kambin Oblique Lateral Lumbar Interbody Fusion With a Low Neurostimulation Threshold and Transient Intraoperative Neuromonitoring Changes Without Postoperative Neurological Deficits

**DOI:** 10.7759/cureus.105724

**Published:** 2026-03-23

**Authors:** Praveen Saxena, Hamid Abbasi, Abdul Rauf, Dominic Moore

**Affiliations:** 1 Spine Surgery, Apollo Hospitals International Limited, Ahmedabad, Ahmedabad, IND; 2 Spine Surgery, Avicenna Technical University (ATU) and Inspired Spine Health, Minneapolis, USA; 3 Neurosurgery, Inspired Spine Health, Minneapolis, USA; 4 Spine Surgery, Inspired Spine Health, Minneapolis, USA

**Keywords:** electromyography, intraoperative neuromonitoring, lumbar spine surgery, oblique lateral lumbar interbody fusion, trans-kambin approach

## Abstract

Trans-Kambin oblique lateral lumbar interbody fusion (OLLIF) is a minimally invasive lumbar fusion technique that relies heavily on intraoperative neuromonitoring (IONM) to reduce the risk of neural injury. Triggered electromyography (tEMG) stimulation thresholds below 3-4 mA are traditionally considered to indicate increased neural risk; however, the clinical implications of isolated low thresholds remain incompletely defined. We report the case of a 44-year-old female who underwent L5-S1 trans-Kambin OLLIF. During probe placement, triggered EMG responses were elicited at 2 mA, indicating close proximity to neural elements. Free-run EMG remained quiet, and baseline motor evoked potentials (MEPs) and somatosensory evoked potentials (SSEPs) were stable during dilation and cage insertion. Approximately 10 minutes after final cage positioning, an ~80% reduction in left L5 MEP amplitude occurred, which persisted until extubation. Despite these transient IONM changes, post-extubation neurological examination was normal, with no motor or sensory deficits. The patient remained neurologically intact at follow-up with improvement in preoperative symptoms. This case demonstrates that isolated low neurostimulation thresholds and transient IONM changes do not inevitably predict postoperative neurological deficit, highlighting the importance of interpreting multimodal neuromonitoring in the clinical context and emphasizing that patient-specific risk-benefit analysis and surgical judgment remain paramount.

## Introduction

Trans-Kambin oblique lateral lumbar interbody fusion (OLLIF) is a minimally invasive lumbar fusion technique that accesses the disc space through Kambin’s triangle while minimizing posterior muscle dissection and soft tissue trauma [1,2]. Due to the close proximity of exiting and traversing nerve roots within this corridor, intraoperative neuromonitoring (IONM) plays a critical role in enhancing procedural safety during trans-Kambin access and instrumentation.

Triggered electromyography (tEMG) is commonly utilized during OLLIF to assess the spatial relationship between surgical instruments or implants and adjacent neural structures. Traditionally, stimulation thresholds below 3-4 mA are considered suggestive of increased neural proximity and potential risk of nerve injury [3-5]. However, the clinical significance of isolated low stimulation thresholds remains incompletely defined, particularly in the absence of sustained neuromonitoring changes or postoperative neurological deficits.

In this report, we describe a case of trans-Kambin OLLIF characterized by low neurostimulation thresholds and transient IONM changes, yet resulting in no postoperative neurological deficit. This case underscores the nuanced role of IONM in minimally invasive lumbar fusion and emphasizes the importance of surgeon experience, anatomical knowledge, and clinical judgment when interpreting neuromonitoring findings.

In this report, the term “isolated low stimulation threshold” refers to a tEMG response occurring at ≤3 mA in the absence of sustained free-run EMG activity, with stable somatosensory evoked potentials (SSEPs), and without persistent or progressive motor evoked potential (MEP) deterioration at the time of access. This operational definition is used to distinguish low threshold proximity alerts from patterns that more clearly indicate ongoing neural irritation or injury.

## Case presentation

A 44-year-old female presented with chronic left-sided low back pain radiating to the left lower extremity, consistent with L5 radiculopathy. Imaging demonstrated degenerative disc disease with foraminal stenosis at L5-S1.

Triggered EMG responses occurred at 2 mA during probe placement. Free-run EMG remained quiet throughout dilation and cage insertion. MEPs and SSEPs were stable during cage placement and final positioning. Approximately 10 minutes after the final cage placement, an ~80% reduction in left L5 MEP amplitude was observed. Contralateral MEPs and all SSEPs remained stable. Hemodynamic and anesthetic parameters were unchanged.

Surgical planning and risk-benefit analysis

The patient was scheduled for minimally invasive trans-Kambin OLLIF at the L5-S1 level following failure of conservative therapy and correlation of symptoms with imaging findings demonstrating degenerative disc disease and foraminal stenosis. Preoperative planning incorporated established OLLIF technical principles emphasizing careful trajectory selection through Kambin’s triangle to minimize neural risk [1,2].

**Figure 1:**
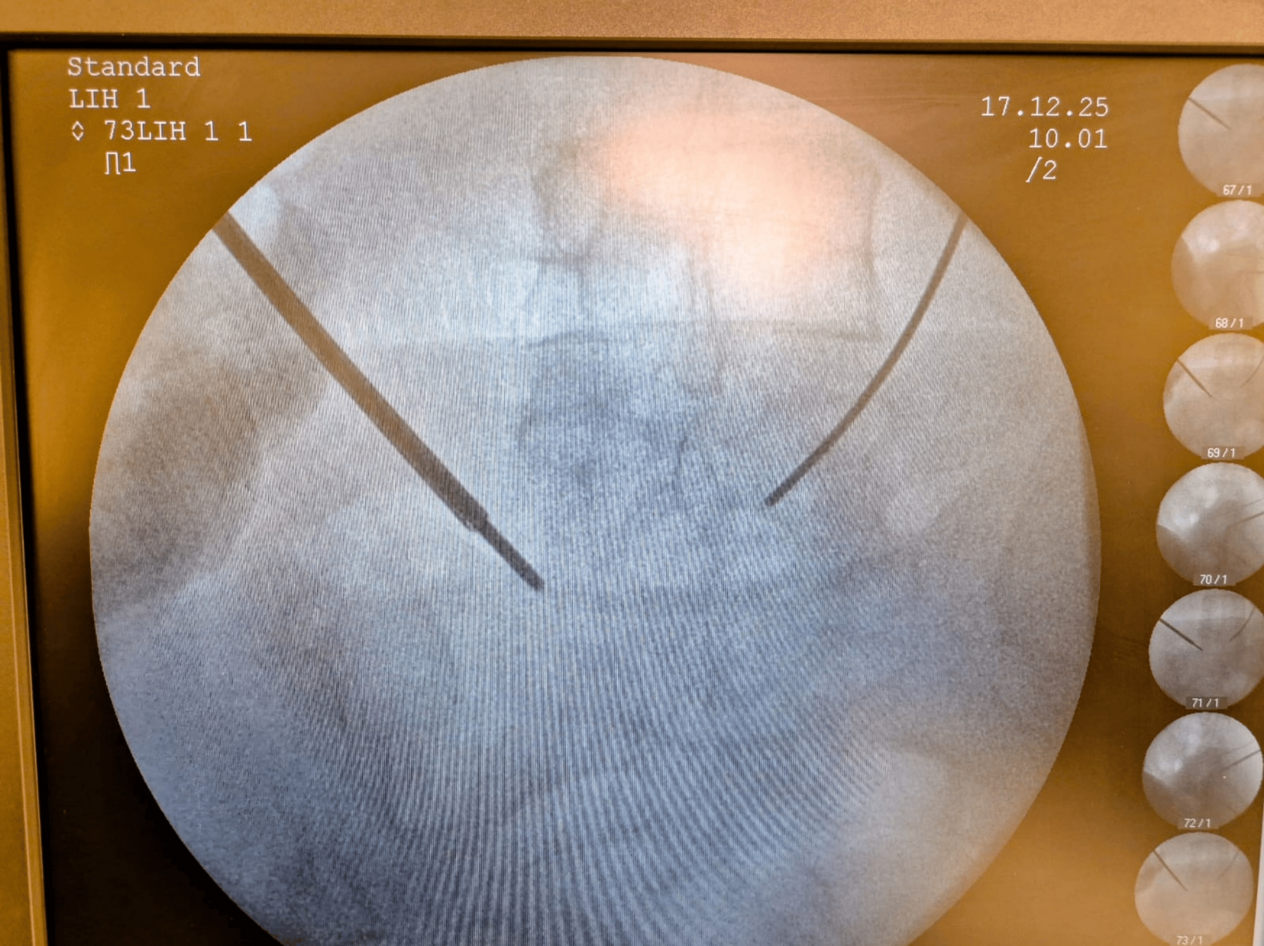
Intraoperative AP imaging of probe placement through Kambin's triangle at 2 mA.

Multimodal IONM was employed, including triggered EMG, free-run EMG, MEPs, and SSEPs, in accordance with established neuromonitoring principles and guidelines [3-7]. During probe placement, triggered EMG responses were elicited at a threshold of 2 mA (seen in Figure 1), indicating closer proximity to neural elements than typically preferred and representing increased theoretical risk [5,6]. Multimodal intraoperative neuromonitoring was performed using a NIM Eclipse 16-channel system. Monitoring modalities included tEMG, free-run EMG, MEPs, and SSEPs.

Conversion to an open procedure was considered; however, it was determined that open conversion would expose the patient to substantially higher morbidity, including increased blood loss, infection risk, soft-tissue injury, and prolonged recovery [1,2]. In the absence of sustained free-run EMG activity, with stable baseline MEPs and SSEPs and favorable patient-specific anatomy, the operating surgeon made an informed decision to proceed with the minimally invasive approach. This decision reflected deliberate real-time integration of neuromonitoring data, anatomy, imaging, and patient-specific risk-benefit considerations rather than reliance on an isolated stimulation threshold.

Reference neuromonitoring parameters and alert thresholds are summarized in Table 1 based on established literature [3-7]. Estimated correlations between triggered EMG thresholds and nerve root distance are outlined in Table 2 [1,2,5,6]. Patient-specific findings and intraoperative timelines are summarized in Table 3 and Table 4, respectively.

**Table 1:** Reference intraoperative neuromonitoring parameters during MIS OLLIF (open vs. MIS comparison) MIS OLLIF: minimally invasive oblique lateral lumbar interbody fusion, EMG: electromyography, MEP: motor evoked potential, SSEP: somatosensory evoked potential

Modality	Parameter	Acceptable range (MIS OLLIF)	Acceptable range (open screws)	Clinical interpretation
Triggered EMG	Screw stimulation	>12–14 mA	≥8–10 mA	Low risk; safe distance from nerve root
Triggered EMG	Screw stimulation	8–12 mA	5–7 mA	Intermediate risk; close proximity to neural elements
Triggered EMG	Screw stimulation	≤8 mA	≤3–4 mA	High risk; likely nerve root proximity
Triggered EMG	Probe stimulation	≥4 mA	≥4 mA	Low risk
Triggered EMG	Probe stimulation	3 mA	3 mA	Intermediate to low risk
Triggered EMG	Probe stimulation	2 mA	2 mA	Unknown risk; insufficient published outcome data
Free-run EMG	Spontaneous activity	None or brief, non-sustained (<60 seconds)	None or brief, non-sustained	Normal finding
Free-run EMG	Sustained neurotonic discharges	Absent	Absent	Suggests nerve irritation if present
MEPs	Global motor pathway monitoring	Not routinely used	Not routinely used	Acceptable for OLLIF
SSEPs	Amplitude change	≤50% decrease	≤50% decrease	Acceptable
SSEPs	Latency change	≤10% increase	≤10% increase	Acceptable

**Table 2:** Triggered EMG thresholds and estimated nerve root distance during OLLIF Note: There are currently no published outcome data defining safety thresholds below 3 mA. EMG: electromyography, OLLIF: oblique lateral lumbar interbody fusion, tEMG: triggered electromyography

tEMG threshold (mA)	Estimated nerve root distance	Risk interpretation
5 mA	~6–7 mm	Very safe
4 mA	~5–6 mm	Safe
3 mA	~4 mm	Intermediate risk
2 mA	<4 mm (estimated)	Unknown risk; subject of current investigation

**Table 3:** Patient-specific neuromonitoring findings EMG: electromyography, MEP: motor evoked potential, SSEP: somatosensory evoked potential

Modality	Finding	Interpretation
Triggered EMG	Initially positive at 2 mA → negative after trajectory adjustment	Close nerve proximity recognized and corrected
Free-run EMG	No sustained neurotonic discharges	No ongoing mechanical nerve irritation
MEPs (Left L5)	~80% transient amplitude reduction	Transient motor pathway disturbance
SSEPs	Stable bilaterally	Preserved sensory pathway integrity

**Table 4:** Timeline of neuromonitoring changes and clinical decisions IONM: intraoperative neuromonitoring, EMG: electromyography, MEP: motor evoked potential, SSEP: somatosensory evoked potential

Surgical step	IONM finding	Clinical action
Probe placement	Triggered EMG initially positive at 2 mA, becoming negative after repositioning	Instrument trajectory adjusted; anatomy re-reviewed; safe corridor confirmed
Dilation	Free-run EMG negative; MEPs and SSEPs stable	Procedure continued with heightened caution
Cage insertion	Triggered EMG negative; free-run EMG quiet; MEPs and SSEPs initially stable	Continued close neuromonitoring observation
Approximately 10 minutes post-placement	Approximately 80% decrease in left L5 MEP amplitude	Hemodynamic status and anesthetic parameters evaluated

Anesthetic course

The procedure was performed under general endotracheal anesthesia. Induction was achieved using propofol (1.5-2 mg/kg) and fentanyl (2 μg/kg), with succinylcholine (2 mg/kg) administered to facilitate intubation.

Anesthesia was maintained using propofol and fentanyl infusion with low-dose sevoflurane (0.6-0.8 MAC). Bispectral index (BIS) monitoring was maintained between 40 and 60 throughout the procedure. Neuromuscular blockade was not maintained during the monitoring phase in order to allow reliable electrophysiologic monitoring.

Postoperative outcome

Immediately following extubation, the patient demonstrated normal motor strength in L5-innervated muscle groups, including tibialis anterior and extensor hallucis longus, with Medical Research Council (MRC) grade 5/5 strength bilaterally. Sensory examination was normal, and no new neurological deficits were identified. Imaging confirmed appropriate cage positioning.

At follow-up, she reported significant improvement in preoperative radicular symptoms and remained neurologically intact. 

## Discussion

This case reinforces that IONM data must be interpreted within a clinical context rather than applied as absolute decision thresholds [3-7]. Multimodal neuromonitoring provides complementary information regarding nerve proximity and functional integrity, but no single parameter should independently dictate intraoperative decision-making.

Prior technical literature on trans-Kambin OLLIF has demonstrated that tEMG thresholds correlate with nerve root proximity, with stimulation thresholds of approximately 3 mA corresponding to an estimated distance of ~4 mm from the nerve root [1,2,4,5]. However, outcome data defining the clinical implications of stimulation thresholds below 3 mA are currently lacking. This case contributes to the emerging body of evidence evaluating the usability and safety considerations of lower stimulation thresholds during OLLIF.

In this case, tEMG played a central role in intraoperative decision-making. The initial low-threshold response at 2 mA alerted the surgical team to close neural proximity, prompting careful adjustment of the probe trajectory. Subsequent negative tEMG responses confirmed a safe corridor, allowing the surgeon to continue the minimally invasive approach with confidence. This demonstrates how real-time interpretation of tEMG, integrated with free-run EMG, MEPs, SSEPs, and anatomical assessment, can effectively mitigate risk and guide safe surgical strategy.

The low stimulation threshold observed in this case was appropriately recognized as a higher theoretical risk but was balanced against the absence of sustained free-run EMG activity, favorable patient anatomy, and the substantially greater morbidity associated with conversion to an open procedure [1,2,6]. These factors supported continuation of the minimally invasive approach with heightened vigilance.

The delayed reduction in MEP amplitude likely represents transient neurapraxia, stretch, or localized ischemia rather than permanent neural injury, as supported by preserved SSEPs and a normal postoperative neurological examination [3,6,7]. Notably, based on extensive prior experience with the OLLIF technique, MEP monitoring has not been routinely required or frequently utilized for this procedure, as described in the original OLLIF technical literature [1]. Similarly, SSEPs have demonstrated limited clinical utility in this surgical context, with large clinical experience indicating that free-run EMG and triggered EMG alone are generally sufficient to perform this procedure safely.

This report does not advocate normalization of low stimulation thresholds. It underscores that experienced surgeons must integrate neuromonitoring findings with anatomy, imaging, and patient-specific factors, with clinical judgment serving as the final determinant of intraoperative management [6,7]. As a single case report, it cannot establish safety thresholds or redefine neuromonitoring alarm criteria, but it illustrates how multimodal monitoring can guide surgical decision-making during minimally invasive lumbar fusion.

## Conclusions

Low neurostimulation thresholds during trans-Kambin OLLIF inherently indicate increased risk and should not be disregarded. However, rigid adherence to isolated neuromonitoring values without contextual interpretation may expose patients to greater harm. Multimodal neuromonitoring should guide, but not replace, experienced clinical judgment.
